# MODY Only Monogenic? A Narrative Review of the *Novel* Rare and Low-Penetrant Variants

**DOI:** 10.3390/ijms25168790

**Published:** 2024-08-13

**Authors:** Iderina Hasballa, Davide Maggi

**Affiliations:** 1 Endocrinology Unit, Department of Internal Medicine and Medical Specialties (DIMI), University of Genoa, 16132 Genoa, Italy; 2 Department of Internal Medicine and Medical Specialties (DiMI), University of Genoa, 16132 Genoa, Italy; 3 Diabetes Clinic, IRCCS Ospedale Policlinico San Martino Genoa, 16132 Genoa, Italy

**Keywords:** maturity-onset diabetes of the young, MODY, monogenic diabetes and diabetes mellitus

## Abstract

Maturity-onset diabetes of the young (MODY) represents the most frequent form of monogenic diabetes mellitus (DM), currently classified in 14 distinct subtypes according to single gene mutations involved in the differentiation and function of pancreatic β-cells. A significant proportion of MODY has unknown etiology, suggesting that the genetic landscape is still to be explored. Recently, *novel* potentially MODY-causal genes, involved in the differentiation and function of β-cells, have been identified, such as *RFX6*, *NKX2.2*, *NKX6.1*, *WFS1*, *PCBD1*, *MTOR*, *TBC1D4*, *CACNA1E*, *MNX1*, *AKT2*, *NEUROG3*, *EIF2AK3*, *GLIS3*, *HADH*, and *PTF1A*. Genetic and clinical features of MODY variants remain highly heterogeneous, with no direct genotype–phenotype correlation, especially in the low-penetrant subtypes. This is a narrative review of the literature aimed at describing the current state-of-the-art of the *novel* likely MODY-associated variants. For a deeper understanding of MODY complexity, we also report some related controversies concerning the etiological role of some of the well-known pathological genes and MODY inheritance pattern, as well as the rare association of MODY with autoimmune diabetes. Due to the limited data available, the assessment of MODY-related genes pathogenicity remains challenging, especially in the setting of rare and low-penetrant subtypes. In consideration of the crucial importance of an accurate diagnosis, prognosis and management of MODY, more studies are warranted to further investigate its genetic landscape and the genotype–phenotype correlation, as well as the pathogenetic contribution of the nongenetic modifiers in this cohort of patients.

## 1. Introduction

Maturity-onset diabetes of the young (MODY) represents the most frequent form of monogenic diabetes mellitus (DM) and accounts for 1–5% of all DM cases [1,2]. The estimated prevalence of MODY, mostly extrapolated from European populations, is approximately 1–5 per 10,000 individuals. Epidemiological data, mainly derived from studies conducted in Europe, North America, and Australia, are suggestive of a certain variability of MODY prevalence in relation to geographical location and ethnicity. To date, the prevalence of MODY in the resting areas, such as Africa, Asia, Middle East, or South America, is not defined yet. Regarding the age of onset, MODY prevalence in adults appears to be 1 per 10,000, whereas in children it is 1 per 23,000 [2]. The diagnostic criteria of the Practice Guideline for MODY in 2008 comprise early onset of diabetes before 25 years of age, history of diabetes in two consecutive generations, absence of pancreatic β-cell autoimmunity, and preserved β-cell function defined by the lack of insulin treatment or serum C-peptide levels > 200 pmol/L after 3 years of insulin therapy [3]. Currently, at least 14 distinct MODY subtypes have been identified according to single-gene mutations inherited by autosomal dominant pattern, and are involved in the differentiation, development, and function of β-cells [4,5]. Such heterogeneity significantly impacts diabetes pathophysiology, clinical course, and treatment response, which highly differ among all MODY forms [6]. Ranging from 80% to 95% of MODY cases, the underlying cause involves the pathogenetic variants of hepatocyte nuclear factor-1 homeobox A (*HNF1A*), hepatocyte nuclear factor-4 homeobox A (*HNF1A*), hepatocyte nuclear factor-1 homeobox B (*HNF1B*), and glucokinase (*GCK*) genes [4,7]. However, despite the molecular advances, a significant proportion of MODY has unknown etiology, which suggests that the genetic landscape is still to be explored. A significant proportion of individuals is labeled as MODY-X, whose genetic diagnosis is unclear [8,9]. Moreover, up to 80–90% of MODY remains underdiagnosed or misdiagnosed as type 1 (T1DM) or type 2 diabetes (T2DM), also due to the highly varied clinical spectrum including overlapping features with T1DM or T2DM [9]. Genetic and clinical characterizations of MODY variants are highly heterogeneous, with no direct genotype–phenotype correlations, especially in the rare and low-penetrant subtypes. Recently, *novel* probable causal genes, involved in the differentiation and function of β-cells, have been detected, such as *RFX6*, *NKX2.2*, *NKX6.1*, *WFS1, PCBD1*, *MTOR*, *TBC1D4*, *CACNA1E*, *MNX1*, *AKT2*, *NEUROG3*, *EIF2AK3*, *GLIS3*, *HADH*, and *PTF1A* [8,10,11,12,13,14,15].

## 2. Novel Rare and Low-Penetrant Genes Associated with MODY

MODY is defined as a single-gene defective diabetes, encompassing highly genetically and clinically heterogenous variants, where genotype is not always predictive of a specific phenotype. To assess the pathogenicity of the causal mutations, several scoring systems have been implemented. Nonetheless, statistical evidence is well established only for some major high-penetrating genes such as *GCK*, *HNF1A*, *HNF4A*, *NEUROD1*, *KCNJ11* and *HNF1B* [16]. In rare MODYs, mutations appear to be associated with reduced penetrance and little pathogenic impact [17]. Therefore, it is plausible that the diversity observed in the penetrance rates of MODY may be likely related to both genetic and nongenetic modifiers [18]. Molecular factors could comprise epigenetic modulation, genetic variant factors, or allele-specific expression patterns. Moreover, similarly to T2DM, other modifiers such as environmental conditions, intrauterine growth, or ethnicity could also contribute to the development of diabetes in this cohort of patients [17]. However, additional studies are warranted to further investigate the molecular mechanisms and the role of nongenetic factors in MODY pathophysiology [10]. Considering the evaluation bias related to clinical assessment, accurate data of prevalence and penetrance regarding rare and low-penetrant MODYs are generally not well known. Furthermore, there is a high prevalence ranging from 46.2 to 73.9% of the so-called MODY-X patients, who fulfill the classic diagnostic criteria for MODY but lack a specific genetic diagnosis [9]. From the limited data available in the literature, MODY-X patients appear to have a highly variable clinical phenotype, which may also be reflective of the heterogeneous genetic background of the studied populations. In a Spanish cohort, MODY-X patients, in comparison with MODY2 or MODY3, presented higher levels of hyperglycemia and reduced insulin sensitivity, whereas, in a French study population, MODY-X individuals displayed a less aggressive clinical course than their counterparts affected by MODY3 [19].

Using the latest DNA sequencing techniques, *novel* genes have been recently identified in MODY cases such as *RFX6*, *NKX2.2*, *NKX6.1*, *WFS1*, *PCBD1*, *MTOR*, *TBC1D4*, *CACNA1E*, *MNX1*, *AKT2*, *NEUROG3*, *EIF2AK3*, *GLIS3*, *HADH*, and *PTF1A* [8,10,11,12,13,14,15]. In these scenarios, rare variants with no direct genotype–phenotype correlations are observed, most likely due to their incomplete penetrance [8].

Information regarding the identities of these genes, their function, and pathophysiology is presented in Table 1.

### 2.1. RFX6 (Regulatory Factor X6)

*RFX6* (6q22.1) encodes RFX6, a component of the RFX family of winged-helix transcription factors involved in pancreatic islet cell differentiation and function [13]. *RFX6*, initially expressed in the endoderm, is limited at mid-gestation to gut and pancreatic bud, and then becomes progressively and ultimately restricted to the endocrine lineage in the mature pancreas [10,20]. Genetic studies in both mice and humans showed that during embryogenesis, RFX6 contributes to directing the β-cell and other islet cells development downstream of the transcription factor neurogenin 3 (NEUROG3), as well as upstream of several islet transcription regulators such as NEUROD1, PAX4, and ARX [20,21]. In addition, RFX6 also participates in modulating insulin secretion in human β-cells by upregulating the expression of the insulin gene and of other genes involved in insulin secretion such as GCK and voltage-dependent calcium channel [22]. Hence, RFX6 may contribute to both pancreatic islet maturation and insulin production in distinct and independent pathways [23]. Furthermore, there is recent evidence that RFX6 also contributes in modulating gene expression and crucial functions of human adult α-cells, comprising glucagon secretion [24]. Biallelic mutations of *RFX6* are associated with Mitchey–Rilley syndrome, a rare autosomal recessive disorder comprising neonatal diabetes, pancreatic hypoplasia, gallbladder agenesis, and duodenal and jejunal atresia. In such context, the severe diabetes phenotype is a consequence of the aberrant pancreatic islet maturation and function, including insulin production [21,23,25]. Heterozygous mutations of *RFX6* and *RFX6* TPV, instead, have been linked to isolated MODY with reduced penetrance [8,26]. In this cohort of patients, diabetes pathogenesis is supported by insulin deficiency associated, however, with normal development of the pancreatic islets [23,27]. Phenotypically, *RFX6* TPV-related MODY was described in the study of Patel et al., enrolling 27 patients, as mild and with a median age at diagnosis of 32 years old. At recruitment, endogenous insulin levels were significant, but insulin treatment was required after 10 years of diabetes in 69% of patients. No relevant sensitivity to sulphonylurea was documented [10]. Moreover, differently from other forms of diabetes comprising T2DM, T1DM, and *HNF1A*-MODY, *RFX6*-MODY was associated with a decreased glucose-dependent insulinotropic peptide (GIP) secretion [10]. In the case report of Imaki et al., regarding a case of MODY caused by a *novel* heterozygous *RFX6* mutation p.R652X, the patient presented, in addition to a reduced insulin and GIP response, a reduced glucagon-like peptide 1 (GLP-1) response. Such findings appear in accordance with the lower GIP and GLP-1 levels detected in *RFX6*-deficient murine models, and may be suggestive of the potential role of GLP-1 receptor agonist therapy in this subset of patients [13]. Furthermore, a good response to dipeptidyl peptidase-4 (DDP-4) inhibitors was also evidenced in other *RFX6*-MODY patients [28]. Therefore, clinical research is necessary to better explore the potential efficacy of GLP-1 receptor agonists and DPP-4 inhibitors in this subset of patients.

### 2.2. NK2.2 (NK2 Homeobox 2)

*NKX2.2* (20p11.22) encodes NKX2.2, a member of the mammalian NK2 homeobox transcriptional regulators, which plays a crucial role in the pancreatic islet cell differentiation, as well as in the morphogenesis of the ventral central nervous system and of the epithelial enteroendocrine cells [29]. Within the pancreas, *NKX2.2* is early expressed in progenitor cells during embryogenesis, and then progressively limited to α-, β-, and pancreatic polypeptide (PP) cells. Murine studies provide evidence of the essential *NKX2.2* involvement not only during the initial islet cell specification, but also in the maintenance of mature β-cell function and in the establishment of proper islet architecture [30]. Consistent with the role of *NKX2.2* documented in mice, in humans, recessive loss-of-function mutations of *NKX2.2* are associated with development delay, neonatal diabetes, and obesity (31 Mio). Regarding obesity, its pathogenesis may be linked to higher levels of ghrelin, an orexigenic hormone, most likely subsequent to the change of pancreatic islet cell differentiation, due to the absent NKX2.2 function, in favor of ghrelin-producing cells rather than the α-, β-, and PP cells [29,31]. Pathogenetic variants of *NKX2.2* have also been detected in extremely rare MODY cases when transmitted by heterozygous inheritance pattern. Thus far, in consideration of the limited data in the literature and of the limited analysis of phenotypes among the family members of these patients, it is difficult to understand precisely the impact of such variants on MODY development, as well as the specific genotype–phenotype correlations [8].

### 2.3. NKX6.1 (NK6 Homeobox 1)

*NKX6.1* (4q21.23) encodes homeobox protein NKX6.1, a transcriptional factor involved in the pancreatic β-cell differentiation and function, as well as in the neural development and motor neuron specification [32,33]. In particular, *NKX6.1* expression is essential both in the initial and late stages of pancreatic development for the specification of multipotent cells (MPCs) into functional β-cell lineage. In the early pancreatic progenitor phase, NKX6.1, especially with PTF1A (pancreas transcription factor 1 α), an important regulator of acinar gene transcription, controls pancreatic cell fate by coordinating the balance in differentiation, proliferation, and maturation of both endocrine and exocrine cells. Moreover, in mature β-cells, NKX6.1 plays a crucial role in the regulation of cell functional properties, including insulin production, glucose uptake and metabolism, and cell proliferation. Despite the fact that in human pancreatic islets NKX6.1 is exclusively limited to β-cells and finalized to maintain cell identity, NKX6.1 also contributes to the suppression of α-cell development by regulating glucagon gene (*GCG*) expression [32,34]. Generally, reduced expression of crucial transcription factors, comprising NKX6.1, is associated with destabilized β-cell homeostasis and function. From accumulating evidence in the literature, decreased levels of NKX6.1 appear to contribute to T2DM pathogenesis both in humans and mice [32,35]. *NKX6.1* deficient β-cells present severely reduced levels of PCSK1 (PC1), which converts proinsulin to insulin, and low expression of zinc transporter Slc30a8 (oxidoreductase Ero1lb), with both mechanisms leading to decreased insulin biosynthesis and secretion [35]. Moreover, aberrant activation of δ-cell genes in *NKX6.1* deficient β-cells could also be observed and contribute furthermore to β-cell identity alterations [32]. An association between *NKX6.1* mutations and MODY development has also been evidenced. In particular, NKX6.1 plays a crucial role in modulating *HNF1A* expression, which is the causative gene of MODY3. Therefore, it is plausible to consider that *NKX6.1* variants could participate in MODY3 pathogenesis by leading to HNF1A-altered levels [36]. Furthermore, heterogeneous pathogenic variants of *NKX6.1* have also been recently associated with extremely rare cases of MODY [8].

### 2.4. WFS1 (Wolframin ER Transmembrane Glycoprotein)

*WFS1* (4p16.1) encodes wolframin, a transmembrane protein of the endoplasmatic reticulum expressed ubiquitously, but mostly in pancreatic β-cells, brain, and heart. Wolframin modulates endoplasmic reticulum calcium homeostasis and calcium signal transduction processes involved in cellular apoptosis [37]. Regarding the pancreas, it is documented that *WFS1* aberrations lead to progressive β-cell loss, altered glucose metabolism, and impaired insulin secretion [38]. There are data in the literature of *WFS1* variants associated with different diabetes phenotypes. Several studies have documented a relevant association of *WFS1* and T2DM, suggesting a possible contribution of the *WFS1* gene in T2DM pathogenesis [39,40,41,42,43,44]. Moreover, a missense alteration of *WFS1* (p.Arg456His) has been also described in one case of T1DM, which could be interpreted as a potential genetic risk factor for T1DM [45]. *WFS1* variants are also responsible for diabetes development in the setting of Wolfram Syndrome 1 (WS1), a rare neurodegenerative disorder with other classical features comprising central diabetes insipidus, optic atrophy, and neurosensorial deafness [37]. WS1 diabetes presents MODY-like features [16,46]. In particular, it is early-onset and nonautoimmune insulin-dependent. WS1 diabetic patients present an average onset age of 6 years old, ranging from 3 weeks to 16 years, and it can be easily misdiagnosed as T1DM. In comparison with T1DM, WS1 patients are usually characterized by inferior values of glycated hemoglobin (HbA1c), lower doses of insulin requirement, fewer cases of ketoacidosis at diagnosis, and a longer remission period [39].

### 2.5. PCBD1 (Pterin-4 Alpha-Carbinolamine Dehydratase 1)

*PCBD1* (10q22.1) encodes a dual-functional protein of the PCBD family, expressed differently in several tissues including the pancreas, and predominantly in the liver and kidney. In the cytoplasm, PCBD1 participates in the tetrahydrobiopterin (BH4) biosynthesis, a cofactor for aromatic amino acid hydroxylases, whereas in the nucleus, it acts as a dimerization coactivator of both HNF1A and HNF1B by enhancing their transcriptional activity, which is crucial for adequate β-cell differentiation and function [47]. Therefore, PCBD1, by stabilizing the above-mentioned HNF1 transcription factors, plays a relevant role both in modulating the progenitor pool during early pancreatic development stages and in maintaining proper homeostasis and function in mature β-cells [12]. Recessive loss-of-function variants of *PCBD1* have been recently associated with early-onset nonautoimmune diabetes with MODY HNF1A-like features, which could benefit from oral antidiabetic treatments such as sulphonylureas or glinides. Thus, in order to optimize the clinical management in this subset of patients, screening for biallelic mutations in *PCBD1* has been suggested in the case of *HNF1A*-like diabetes occurrence with absent alterations in *HNF1A* and *HNF1B*. Heterozygous variants of *PCBD1*, instead, may participate in the development of type 2 diabetes, particularly when present in addition to other risk factors such as excess weight and age [12]. More studies are warranted to further investigate the pathogenetic mechanisms of *PCBD1*, the associated diabetes clinical course, and the response to treatment.

### 2.6. MTOR (Mechanistic/Mammalian Target of Rapamycin)

*MTOR* (1p36.22) encodes mTOR protein, a ubiquitous serine/threonine kinase member of the PI3K-associated protein kinases (PIKK) family. mTOR is a catalytic component of two distinguished complexes MTORC1 and MTORC2, which are involved in a plethora of crucial functions modulating cellular survival, growth, metabolism, and proliferation. In response to different stimuli including growth factors, nutritional inputs, and energetic and oxygen intracellular levels, mTOR regulates key pathways in the biogenesis of proteins, lipids, and nucleotides, as well as in the repression of cell catabolism and autophagy. Several studies have unveiled the role of mTOR in β-cell function and glucose homeostasis since embryogenesis; however, to date, it remains inconclusive whether the mTOR pathway positively or negatively impacts diabetes pathogenesis [48,49]. mTOR activation may enhance β-cell proliferation and insulin secretion with subsequent reduction in glycemia, thus protecting against diabetes. On the other hand, a protracted hyperactivation of mTOR may lead to elevated blood glucose levels due to the depletion of insulin secretory capacity and increased β-cell death [50]. Moreover, chronic activation of mTOR may also alter the metabolic and functional properties of specific immune cells, hence contributing to β-cell impairment and diabetes development [50]. In addition to the complexity of mTOR-diabetes association, the inhibition of mTOR, as a result of oncological target therapies, is also associated with the onset of hyperglycemia or DM [14]. Recently, in a Polish cohort, two variants of mTOR (p.Gln2063ArgfsTer3 and p.Phe871Cys) were found in two subjects affected by MODY-X, in exclusive dietary and behavioral treatment, with positive family history for DM, normal levels of fasting C-peptide, nonelevated HbA1c at diagnosis, and absence of pancreatic islet autoimmunity. The demonstrated familial cosegregation of p.Gln2063ArgfsTer3 with diabetes may suggest variant pathogenicity and a potential association of *MTOR* with rare MODY cases [14].

### 2.7. TBC1D4 (TBC1 Domain Family Member 4)

*TBC1D4* (13q22.3) encodes TBC1D4, also known as AS160, a Rab-GTPase-activating protein (Rab GAPs), which plays a significant role in modulating the insulin-induced glucose transport into skeletal muscle and adipose tissue. In particular, insulin triggers a phosphorylation cascade of events, including PI3K/Akt, that activate several downstream effectors comprising TBC1D4. The phosphorylated TBC1D4 promotes glucose transporter type 4 (GLUT4) translocation to the plasma membrane, thus increasing glucose uptake into adipocytes and muscle cells [51]. An impaired function of TBC1D4 appears to be involved in insulin resistance and T2DM development. Such association was observed in homozygous carriers of a nonsense variant of *TBC1D4* (p.Arg684Ter) in a Greenlandic population. The heterozygous carriers, instead, presented only a slight increase in the 2 h glycemia after the oral glucose intolerance test (OGTT) [14]. A *TBC1D4* heterozygous variant c2T>C (p.Met1?), already classified as likely pathogenic according to the American College of Medical Genetics and Genomics (ACMG) guidelines, was reported in one patient of a Polish cohort of MODY-X individuals. The patient carrying such a variant presented an early onset diabetes diagnosed at 8 years old and underwent insulin treatment. Jakiel et al. suggested that rare heterozygous variants with a major impact could contribute to MODY pathogenesis, while those with less impact to T2DM. However, currently, no direct causative link between such variant and MODY can be certainly demonstrated [14].

### 2.8. CACNA1E (Calcium Voltage-Gated Channel Subunit Alpha1 E)

*CACNA1E* (1q25.3) encodes Cav2.3 protein, an R-type (or initially called “E-type”) high-voltage-gated calcium channel belonging to the voltage-gated CA^2+^ channel family, predominantly expressed in neuronal and neuroendocrine cells [52]. Cav2.3 regulates Ca^2+^ influx into excitable cells in response to cellular membrane depolarization. Cav2.3 is involved in a myriad of calcium-dependent processes including hormone and neurotransmitter release, muscle contraction, cell death, and gene expression [53]. In particular, Cav2.3 plays a significant role in glucose homeostasis since it modulates second-phase insulin secretion, controlling the exocytosis of insulin-containing vesicles with subsequent hormonal release by β-cells. Moreover, Cav2.3 may also regulate the hormonal release of α- and δ-cells, as well as contribute to the differentiation of the mature islet cell lineages via mechanisms not fully elucidated [54]. In Cav2.3−/− murine models, fasting hyperglycemia has been observed not only due to impaired late-phase insulin secretion, but also to an altered glucagon release. Such an effect is supposed to be indirectly caused by a lower secretion of somatostatin, which exerts an inhibitory paracrine effect on both insulin and glucagon release. Several *CACNA1E* polymorphisms have been associated with an increased risk of T2DM [54]. In two MODY-X patients, *novel* variants of *CACNA1E* were identified, p.Tyr1469His and p.Ala324Asp, both of them defined as variants of uncertain significance (VUS) according to ACMG guidelines. The patient carrying the first variant developed impaired fasting glucose (IFG) at the age of 31 years and underwent dietary treatment, while the patient carrying the second variant developed diabetes at the age of 6 months, requiring insulin therapy. Similarly to *TBC1D4*, Jakiel et al. hypothesized that rare heterozygous variants of *CACNA1E* with a major detrimental effect may be associated with monogenic diabetes, whereas homozygous variants with a smaller impact with T2DM [14].

### 2.9. MNX1 (Motor Neuron and Pancreas Homeobox 1)

*MNX1* (7q36.3) encodes a homeobox protein (previously known as Hb9 or Hlxb9), which plays a critical role as a transcriptional factor in the differentiation and development of spinal cord motor neuron cells and pancreatic islet cells. In particular, *MNX1* is involved during embryogenesis in the dorsal pancreatic bud formation, differentiation, and proliferation of β-cells [55]. Heterozygous loss of function *MNX1* variants present the underlying genetic cause of Currarino syndrome, a rare condition characterized by the classic triad of a presacral mass (an anterior meningocele, a teratoma, or an enteric cyst), anterior sacral bone anomaly (sickle-shaped sacrum or complete sacral agenesis), and anorectal malformations [56]. Rare cases of homozygous *MNX1* mutations have been associated, instead, with permanent neonatal diabetes mellitus [55]. A *novel* variant p.His196_Ala198del, formerly reported as VUS, was found in one patient with MODY-X in a heterozygous inheritance pattern. Like *GCK* and *RFX6*, homozygous variants of *MNX1* could contribute to neonatal diabetes development, while heterozygous ones to rare cases of MODY [14]. *MNX1* was reported as a rare gene potentially associated with MODY also in a Southern Indian population [11].

### 2.10. AKT2 (AKT Serine/Threonine Kinase 2)

*AKT2* (19q13.2) encodes the serine/threonine kinase Akt, also known as protein kinase B (PKB β), a crucial enzyme involved in the insulin-signaling pathway, in the modulation of glucose, and lipid metabolism. The expression of *AKT2* during embryogenesis is predominantly high in insulin-sensitive organs, i.e., liver, skeletal muscle, and brown fat, but it is also expressed in the cardiac, cerebral, bone marrow, small intestine, and renal tissue. In response to the insulin receptor activation in the target cells, AkT2 modulates the glucose uptake and metabolism, as well as enhances FoxO3 signaling pathway, which supports β-cell function and regeneration [57]. There is evidence that *AKT2* ablation in mice leads to hyperglycemia, hyperinsulinemia, insulin resistance, and diabetes development [58]. In humans, variants in *AKT2* that lead to a deficient insulin signaling have also been associated with a major risk of insulin resistance and T2DM [59]. Additionally, some rare loss-of-function alterations of *AKT2* have been reported in monogenic forms of diabetes, and may also play a role in MODY development [11].

### 2.11. NEUROG3 (Neurogenin 3)

*NEUROG3* (10q22.1) encodes a basic helix-loop-helix transcription factor, which plays a central role in modulating endocrine cell differentiation in the pancreas and intestine, as well as the expression of NEUROD1 that is already associated with MODY6. *NEUROG3* homozygous mutations have been reported in neonatal diabetes and malabsorptive diarrhea [15,60,61]. A *novel* mutation (p.Arg55Glufs*23) was identified in one female patient of a Chinese cohort affected by MODY. The age at diagnosis was 14 years old, and the clinical status was characterized by hyperglycemia and mild intermittent abdominal pain [15].

### 2.12. Additional Rare Gene Variants Potentially Associated with MODY

Other rare variants found in a South Indian population that could potentially play a role in MODY pathogenesis are *EIF2AK3*, *GLIS3*, *HADH*, and *PTF1A* [11]. Such genes have already been associated with other forms of diabetes. Additional studies are necessary to provide further genetic and functional evidence regarding their contribution to MODY development.

#### 2.12.1. *EIF2AK3* (Eukaryotic Translation Initiation Factor 2-Alpha Kinase 3)

*EIF2AK3* (2p11.2), also known as protein kinase R (PKR)-like endoplasmic reticulum kinase (*PERK*), is involved in fetal β-cell differentiation, function, and proliferation, as well as in the formation of the pancreatic islet architecture. *PERK* deficiency has been associated with neonatal diabetes in the setting of Wolcott–Rallison syndrome (WRS), likely due to embryonic defects in the development of β-cells that lead to β-cell dysfunction or reduced β-cell mass [62].

#### 2.12.2. *GLIS3* (Glis Family Zinc Finger Protein 3)

*GLIS3* (9p24.2) encodes GLIS3 protein, a crucial transcription factor involved in β-cell development and maturation. While *GLIS3* deletion has been associated with neonatal diabetes, single-nucleotide polymorphisms have been reported both in T1DM and T2DM [63].

#### 2.12.3. *HADH* (3-Hydroxyacyl-CoA Dehydrogenase)

*HADH* (4q25) encodes the enzyme 3-hydroxyacyl-CoA dehydrogenase, which plays a critical role in fatty acid beta-oxidation [64]. *HADH* mutations have been identified in infants with hyperinsulinemic hypoglycemia; however, the specific underlying mechanisms are not completely clear [65,66].

#### 2.12.4. *PTF1A* (Pancreas Transcription Factor 1, Alpha Subunit)

*PTF1A* (10p12.2) encodes a protein subunit of the pancreas transcription factor 1 complex, which is involved in the early and late-stage pancreas development and differentiation of both endocrine and exocrine cells. *PTF1A* mutations have been reported in cerebellar and pancreatic agenesis, in neonatal diabetes, and in pancreatic ductal adenocarcinoma [67].

## 3. Controversial Data of MODY-Related Genetic Background

In the literature, there are ambiguous data about the etiological role of several genetic alterations currently linked to MODY. In particular, some variants such as *PDX1* (Pancreatic Duodenal Homeobox 1) variant P33T, E224, P242L (MODY4), *HNF4A* V169I (MODY1), *BLK* A71T (MODY11), and *NEUROD1* (Neurogenic Differentiation 1) H241Q (MODY6) were reported in the study of Mohan et al. to have almost no impact in diabetes development. In fact, their prevalence in both MODY patients and the general population of South India was similar [11]. The controversy could be attributed to the limited population-specific data and, subsequently, to its challenging interpretation. In addition, considering the distinct diabetes phenotype in the Southern Asian population, a different pattern of gene variation and inheritance of MODY may be considered plausible in this cohort of patients [11].

Furthermore, a reanalysis of published variants of *BLK* (MODY11), *PAX4* (Paired Box Gene 4, MODY9), and *KLF11* (Krüppel-Like Factor 11, MODY7) failed to demonstrate variant- and gene-level evidence of a causal correlation between these genes and MODY. Thus, based on these results, it was suggested not to include *BLK*, *PAX4,* and *KLF11* in the diagnostic genetic panels [1]. Accordingly, another study conducted on a European cohort did not document any relevant linkage between *BLK* gene and MODY development. *BLK* mutations, instead, resulted as slightly associated with T2DM, especially in obese individuals [68]. Additionally, there is evidence in the literature supporting the role of some of the abovementioned genes not only in MODY pathogenesis, but also in other forms of diabetes. The underlying molecular mechanisms are still to be investigated. In particular, common variants of *KCNJ11* (Potassium Inwardly Rectifying Channel Subfamily J Member 11) and *ABCC8* have been related to a major susceptibility to T2DM and neonatal diabetes [69]. *KCNJ11* and *ABCC8* mutations both determine a compromised insulin secretory response. The underlying mechanism in the first case is the reduction in K_ATP_ channel sensitivity to ATP, while in the latter it is the membrane hyperpolarization caused by Mg-nucleotide binding to nucleotide-binding domains of SUR1 [70,71,72]. In addition, more than 90% of MODY patients carrying *KCNJ11* or *ABCC8* pathogenetic variants present a favorable response to oral treatment with sulfonylureas [69]. Similarly, mutations of *PDX1* gene (MODY4), which plays a crucial role in β-cell differentiation and function, are also associated with neonatal diabetes and T2DM, as well as ketosis-prone diabetes (KPD) [73,74,75,76]. Also, some *PAX4* (MODY9) pathogenic variants have been linked to T2DM and KPD. In the patients carrying such variants, there is evidence of insulin deficiency due to the impairment of β-cell development and hyperglucagonemia subsequent to the altered transcriptional *PAX4* repression of glucagon [77,78,79]. Polymorphisms or mutations of *NEUROD1* (MODY6), essential for the development and maintenance of mature β-cell activity, were also reported in individuals affected by permanent neonatal diabetes and T2DM [80,81]. *INS* (preproinsulin gene) mutations, which cause hyperinsulinemia by promoting the production of structurally defective insulin, have been linked to T1DM and T2DM, and neonatal diabetes other than MODY10 [82,83]. Furthermore, variants of the most common causal MODY genes *HNF1A*, *HNF4A*, *HNF1B,* and *GCK* have been found to be associated with an increased predisposition of T2DM in several populations [84,85,86,87].

Moreover, a different inheritance pattern of MODY may also be considered, especially if low-penetrant genes are involved. One case of compound heterozygosis was described by Ivanoshchuk et al., who identified the coinheritance of variant c.1562G>A (p.Arg521Gln) in the ABCC8 gene (ATP-binding cassette transporter subfamily C member 8) and c.160C>T (p.Arg54*) in the *HNF1A* gene. Ivanoshchuk et al. described a patient carrying both the c.1562G>A (p.Arg521Gln) variant in the *ABCC8* gene (ATP-binding cassette transporter subfamily C member 8) and the c.160C>T (p.Arg54*) substitution of *HNF1A* gene, respectively, associated with MODY12 and MODY3 in a diabetic patient and his mother in Western Siberia. While c.160C>T in *HNF1A* is already defined as pathogenic, c.1562G>A in *ABCC8*, instead, is characterized by “conflicting interpretations of pathogenicity” or “uncertain significance” [88].

Last, but not least, the complexity and remarkable heterogeneity of this form of diabetes are also highlighted by the extremely rare cases of the coexistence of MODY and positive pancreatic autoimmunity reported in the literature. Such presentations appear intriguing since, currently, the absence of islet cell autoantibodies represents one of the pivotal criteria for MODY identification and subsequent genetic testing. The rare association between MODY and autoimmune diabetes may occur simultaneously or consecutively. In particular, such cases evidence the need to evaluate the presence of autoantibodies in MODY patients, especially when glycemic control declines unexpectedly [89].

## 4. Molecular Advances and Future Prospectives

Since the first description of MODY cases in the 1960s and the coining of the “MODY” acronym in 1974, there have been remarkable advances in our understanding of the maturity-onset diabetes of the young spacing from phenotypic spectrum and therapeutic approaches to molecular diagnostics [90,91]. In particular, after the first genetic mutations identified in the 1990s regarding *GCK* (MODY2), *HNF1A* (MODY3), and *HFN4A* (MODY1), the development of DNA sequencing methods has significantly enhanced the exploration of *novel* causal variants [6,9,92,93,94,95]. Sanger sequencing represents the conventional genetic testing for single-gene genetic disorders, leading, however, to a relevant rate of inconclusive MODY diagnosis (also known as MODY-X) ranging from 46.2 to 73.9% [9]. Unlike the candidate gene approach based on the Sanger method, the advent of next-generation sequencing (NGS) technologies, such as whole-genome sequencing, whole-exome sequencing, and targeted sequencing, currently offer a pivotal role in providing valuable insights into the genetic architecture of MODY. By providing a highly sensitive and specific technique, NGS enables the detection of both common and rare pathogenetic variants associated with the maturity-onset diabetes of the young [9,96]. Additionally, the efficacy of NGS is also highlighted by its accelerated turnaround time of comprehensive genetic profiling, which has expedited the diagnostic algorithm, subsequently enabling a prompt and tailored clinical management of the patient. The implementation of NGS is also essential for the genetic counseling of offspring and relatives, which provides valuable information regarding prognosis and screening of potential complications, as well as informed decisions about diabetes therapeutic strategies or lifestyle interventions in some specific subgroups of MODY or in asymptomatic mutation carriers. Furthermore, such evaluation could also help in better understanding the inheritance pattern, especially in the rare and low-penetrant variants and in different populations that present a highly variable genetic background. Despite the increasing efficiency of NGS-based approaches in identifying DNA variants, and the continuous development and improvement of annotation tools, the validation by functional studies is still a mandatory requirement in the presence of variants of uncertain pathogenic significance, particularly when their knowledge is applied to guide the clinical management of the patient and genetic counseling to the family. In the era of precision medicine, NGS affordability, however, differs widely among diverse sociogeographical settings, also due to financial considerations [97]. According to the 2008 Practice Guidelines for genetic diagnosis of MODY, molecular analysis is recommended only for individuals fulfilling the clinical criteria of MODY [3]. Therefore, the limitations of the advanced genetic investigations, as well as the high prevalence of MODY misdiagnosis, have enhanced the exploration of biomarkers aimed at optimizing diagnostic accuracy [6]. Bonner et al. documented a correlation between the overexpression of miR-103 and miR-224 and *HNF1A*-MODY, suggesting that the detection of such miRNAs in the serum of *HNF1A*-MODY patients could be useful for differential diagnosis [98]. Alternative biomarkers could also be epigenetic signatures, such as DNA methylation or histone acetylation, of genes impacting β-cell development and function. It was documented that aberrant DNA methylation patterns, which mediate a major risk of diabetes in subjects exposed to a diabetic intrauterine environment, concern promoter regions of genes associated with MODY, other than with T2DM and the NOTCH signaling pathway [99]. Indeed, accumulating evidence in epigenetics could provide a deeper understanding of the complex interplay between genetic and environmental factors in MODY pathogenesis. Furthermore, the identification of potential epigenetic biomarkers could represent an exciting prospect for advancing precision medicine for both the diagnosis and management of MODY patients, finally offering a more effective and personalized therapy, including any future implementation of epigenetic targeted strategies such as demethylating agents or histone deacetylase inhibitors in the affected individuals.

## 5. Conclusions

Due to the limited data available, the small-scale population-specific studies, and the unclear causal mechanisms of a significant proportion of MODY cases, the assessment of the related genes pathogenicity remains challenging, especially in the context of rare and low-penetrant variants. Monogenic and polygenic diabetes classically represent two distinguishable conditions. The first one is generally associated with single high-penetrating gene mutations, whereas the latter with the contribution of both several genetic and environmental factors. The controversial data regarding the abovementioned genes, including the identification of other inheritance patterns, underscore the relevant variability of MODY and the difficult interpretation of the specific pathogenetic contribution, in particular of the rare and reduced-penetrant genetic variants. Therefore, it could be potentially reasonable to question the definition of MODY as exclusively monogenic, particularly in this subset of rare and peculiar cases.

In conclusion, more studies are warranted to further explore the genetic landscape of MODY, its correlation with the phenotype, and the impact of the nongenetic modifiers in the diabetes pathophysiology, considering the crucial importance of an accurate diagnosis and treatment in this cohort of patients. Also, the genetic counseling for the offspring and family members plays a pivotal role in providing valuable insights into the underlying genetic mechanisms of MODY. The implementation of NGS as well as further advances in epigenetic research could lead to accurate and early diagnosis, and, subsequently, to tailored therapeutic approaches for the patients in the precision medicine era.

## Figures and Tables

**Table 1:** Identity and function of *novel* rare genes potentially associated with MODY.

Gene Symbol (Gene Name)	OMIM ID	Locus	Function	Clinical and Laboratory Characteristics
*RFX6* (Regulatory Factor X6)	612659	6q22.1	Regulates pancreatic islet cell differentiation and function, including β-cell insulin and α-cell glucagon secretion.	*RFX6* TPV-related MODY Mild phenotype. Median age at diagnosis 32 yo. Eventual requirement of insulin therapy. No relevant sensitivity to sulphonylurea. Potential role of GLP1-RA and DPP4i. Progressive insulin deficiency. Reduced GIP and GLP-1 response.
*NK2.2* (NK2 Homeobox 2)	604612	20p11.22	Modulates pancreatic islet cell differentiation during embryogenesis and mature β-cell function, regulates pancreatic islet architecture. Participates in the morphogenesis of the ventral central nervous system and of the epithelial enteroendocrine cells.	No specific genotype–phenotype/laboratory correlations due to limited data in the literature.
*NKX6.1* (NK6 Homeobox 1)	602563	4q21.23	Regulates pancreatic β-cell differentiation and function, including insulin production, glucose uptake and metabolism, and cell proliferation. Regulates glucagon gene (*GCG*) expression, may induce suppression of pancreatic α-cell development. Contributes to the neural development and motor neuron specification.	No specific genotype–phenotype/laboratory correlations due to limited data in the literature.
*WFS1* (Wolframin ER Transmembrane Glycoprotein)	606201	4p16.1	Modulates endoplasmic reticulum calcium homeostasis and calcium signal transduction processes involved in cellular apoptosis. Regulates β-cell function, glucose metabolism and insulin secretion.	Average age at diagnosis 6 yo (range 3–16 yo). Insulin-dependent (lower doses of insulin requirement than T1DM). Fewer cases of ketoacidosis at diagnosis than T1DM. Longer remission period than T1DM. Inferior values of HbA1c than T1DM. Negative pancreatic autoimmunity.
*PCBD1* (Pterin-4 Alpha-Carbinolamine Dehydratase 1)	126090	10q22.1	In nucleus acts as a dimerization cofactor of HNF1A and HNF1B, and enhances HNF1A/B-mediated transcription. In cytoplasm modulates the tetrahydrobiopterin (BH4) biosynthesis.	MODY *HNF1A*-like features. Potential benefit from oral antidiabetic treatments such as sulphonylureas or glinides.
*MTOR* (Mechanistic Target of Rapamycin)	601231	1p36.22	Antidiabetic effects: mTOR activation may enhance β-cell proliferation and insulin secretion with subsequent reduction in glycemia. Prodiabetic effects: A protracted hyperactivation of mTOR may lead to elevated blood glucose levels due to the depletion of insulin secretory capacity and increased β-cell death. Chronic activation of mTOR may alter the metabolic and functional properties of specific immune cells, hence contributing to β-cell impairment.	No specific genotype–phenotype/laboratory correlations due to limited data in the literature. Found in 3 Polish patients with MODY-X: Age at diagnosis 8–24 yo. Dietary intervention.
*TBC1D4* (TBC1 Domain Family Member 4)	612465	13q22.2	Modulates glucose transporter type 4 (GLUT4) translocation to the plasma membrane with subsequent control of glucose uptake into adipocytes and muscle cells.	No specific genotype–phenotype/laboratory correlations due to limited data in the literature. Found in one Polish patient with MODY-X Age at diagnosis 8 months. Insulin therapy.
*CACNA1E* (Calcium Voltage-Gated Channel Subunit Alpha1 E)	601013	1q25.3	Regulates Ca^2+^ influx into excitable cells and is involved in physiological calcium-dependent processes. Modulates second-phase insulin secretion by β-pancreatic cells. Regulates the release of glucagon and somatostatin by α- and δ- pancreatic cells. Contributes to the differentiation of the mature pancreatic islet cell lineages via mechanisms not fully elucidated.	No specific genotype–phenotype/laboratory correlations due to limited data in the literature. Found in 2 Polish patients with MODY-X. (I) Patient with IFG at diagnosis: age at onset 31 yo, dietary intervention. (II) Patient with DM at diagnosis: age at onset 6 months, insulin therapy.
*MNX1* (Motor Neuron and Pancreas Homeobox 1)	142994	7q.36.3	Regulates the differentiation and development of spinal cord motor neuron cells and pancreatic islet cells, in particular β-cells.	No specific genotype–phenotype/laboratory correlations due to limited data in the literature. Found in 1 Polish patient with MODY-X Age at diagnosis: 53 yo. Insulin therapy.
*AKT2* (AKT Serine/Threonine Kinase 2)	164731	19q13.2	Modulates the glucose uptake and metabolism in insulin target cells. Promotes FoxO3 signaling pathway, which supports β-cell function and regeneration.	No specific genotype–phenotype/laboratory correlations due to limited data in the literature.
*NEUROG3* (Neurogenin 3)	604882	10q22.1	Regulates endocrine cell differentiation in the pancreas and intestine. Regulates the expression of *NEUROD1* (associated with MODY6).	No specific genotype–phenotype/laboratory correlations due to limited data in the literature Found in 1 Chinese patient with MODY-X: Age at diagnosis: 14 yo. Hyperglycemia and mild intermittent abdominal pain.
*EIF2AK3* (Eukaryotic Translation Initiation Factor 2-Alpha Kinase 3)	604032	2p11.2	Modulates fetal β-cell differentiation, function and proliferation. Regulates the development of the pancreatic islet architecture.	No specific genotype–phenotype/laboratory correlations due to limited data in the literature.
*GLIS3* (Glis Family Zinc Finger Protein 3)	610192	9p24.2	Encodes a crucial transcription factor involved in β-cell development and maturation.	No specific genotype–phenotype/laboratory correlations due to limited data in the literature.
*HADH* (3-Hydroxyacyl-CoA Dehydrogenase)	601609	4q25	Plays a critical role in fatty acid β-oxidation.	No specific genotype–phenotype/laboratory correlations due to limited data in the literature.
*PTF1A* (Pancreas Transcription Factor 1, Alpha Subunit)	607194	10p12.2	Regulates the early and late-stage pancreas development and differentiation of both endocrine and exocrine cells.	No specific genotype–phenotype/laboratory correlations due to limited data in the literature.

Abbreviations: *RFX6* TPV: RFX6 protein-truncating variant, *yo*: years old, *GLP-1 RA*: Glucagon-like peptide-1 receptor agonists; *DPP4i*: Dipeptidyl peptidase 4 inhibitors, *GIP*: glucose-dependent insulinotropic peptide, *GLP-1*: glucagon-like peptide 1, *T1DM*: type 1 diabetes mellitus, *HNF1A*: hepatocyte nuclear factor-1 homeobox A, *HNF1B*: hepatocyte nuclear factor-1 homeobox B, *MODY*: maturity-onset diabetes of the young, IFG: impaired fasting glucose, *HbA1c*: glycated hemoglobin, *OMIM:* The Online Mendelian Inheritance in Man.
